# High‐intensity interval training improves performance in young and older individuals by increasing mechanical efficiency

**DOI:** 10.14814/phy2.13232

**Published:** 2017-04-05

**Authors:** Georges Jabbour, Horia‐Daniel Iancu, Pascale Mauriège, Denis R. Joanisse, Luc J. Martin

**Affiliations:** ^1^ School of Kinesiology and LeisureFaculty of Health Sciences and Community ServicesUniversité de MonctonMonctonNew BrunswickCanada; ^2^ Department of KinesiologyUniversité LavalQuébecCanada; ^3^ Department of BiologyFaculty of SciencesUniversité de MonctonMonctonNew BrunswickCanada

**Keywords:** Energy expenditure, mechanical efficiency, power, ratings of perceived‐exertion, ventilator thresholds 1, ventilator thresholds 2

## Abstract

This study evaluated the effects of 6 weeks of high‐intensity interval training (HIIT) on mechanical efficiency (ME) in young and older groups. Seventeen healthy young adults [26.2(2.4) year], and thirteen healthy older adults [54.5(2.3) year] completed a 6‐week HIIT intervention (three sessions per week) on an electromagnetically braked cycle ergometer. Each HIIT session contained six repetitions of supramaximal exercise intervals (6 seconds each) with 2 min of passive recovery between each repetition. ME (%) were computed in net terms across stages corresponding to ventilator thresholds 1 (VT1) and 2 (VT2) and at 100% of maximal oxygen consumption (*V*O_2_max) of an incremental maximal cycling test. After 6 weeks, the ME values did not differ between the two groups and were significantly higher than the ones at baseline (*P *<* *0.01). In this study, the multiple linear regression analysis demonstrated the increases in maximal power (Pmax) contributed significantly to ME increases over 6 weeks at VT1, VT2 and at 100% of VO_2max_. This model accounted respectively for 28, 38, and 42%, of the increases. In older adults, ME determined during incremental maximal cycling test increases at VT1, VT2 and at 100% over 6‐week HIIT intervention, and the increment appeared to be related to increases in Pmax. HIIT can be recommended as a strategy aimed at improving muscle efficiency among older adults.

## Introduction

It has been shown that reduced physical performance in older adults is associated with increased mortality (Studenski et al. 2011). In fact, aging involves reductions in muscle mass (Marcell 2003), changes in motor unit morphology, and impaired motor performance (Hunter et al. 2001). Such limitations reduce maximal strength and power, slow contractile velocity, and increase fatigability in this population.

Another aspect to consider is the increase in metabolic energy consumption in older (greater than 65 year of age) adults that could lead to impaired performance (Martin et al. 1992; Hortobágyi et al. 2011). In fact, studies have revealed that increase in metabolic costs likely lead to muscle fatigue during walking and can contribute to lower participation in walking exercise in older adults. Indeed, to walk a given distance, several studies (Martin et al. 1992; Hortobágyi et al. 2011) reported high‐energy expenditure values in older compared to young adults. Consequently, the metabolic cost of performing mechanical work, that is, mechanical efficiency, represents a key determinant of performance in older adults (74.5 ± 2.9 years) (Ortega and Farley 2015). Mechanical efficiency refers to the amount of work performed for a given energy consumption; stated differently, the ability of an individual to convert energy consumed into external work (Weinstein et al. 2004). Studies have shown that elderly adults (up to 65 years) perform mechanical work 6–17% less efficiently than young adults (Mian et al. 2006; Woo et al. 2006), and both sedentary and healthy elderly adults consume ∼15–30% more metabolic energy to transport a kilogram of body mass a given distance than do young adults (Pearce et al. 1983; Ortega and Farley 2007). While most evidence comes from walking studies, others have calculated exercise efficiency during bicycle ergometer testing and shown that elderly adults have similar (Babcock et al. 1992), greater (Venturelli et al. 2013), or lower (Bell and Ferguson 2009) exercise efficiency than young adults. The discrepancy between these studies may be related to the protocols used to collect the data. In fact, several studies that showed similar or greater exercise efficiency in the aged population used a non‐steady‐state “ramp” protocol, where metabolic energy expenditure for each mechanical power level was quantified from the rate of oxygen consumption during a single minute at each power level (Babcock et al. 1992; Venturelli et al. 2013). Since it is unlikely that older subjects reach metabolic steady state within a minute, it is possible that this technique underestimates metabolic energy expenditure and overestimates efficiency (Bell and Ferguson 2009; Venturelli et al. 2013). In contrast, Bell and Ferguson (2009) quantified metabolic energy expenditure and exercise efficiency during steady‐state cycle ergometer and found that healthy elderly women (~70 years) performed mechanical work with ∼20–30% lower net efficiency [total work/(total cost − resting cost)] than healthy young women. Using the same steady‐state calculation of exercise efficiency during walking, Ortega and Farley (2015) reported a lower net efficiency of mechanical work in older compared to younger adults across a wide range of walking speeds. Considering that an older population can exhibit greater metabolic cost during exercise, evaluating ME may be valuable with respect to the detection of muscle dysfunction and to assess any subsequent adaptations in response to training.

In terms of interventions among older adults, high‐intensity interval training (HIIT) has been reported as a valuable strategy for improving indices of performance, as well as several health parameters even over brief periods (Gillen and Gibala 2013). While several studies have reported lower ME in older compared to younger adults, no study has evaluated ME adaptations following an intervention program in this population. Studies in young adults have reported significant increases in ME in response to power training (Kyröläinen et al. 2004) as well as to HIIT (Gendron et al. 2016). Considering that HIIT demonstrated a multitude of physiological adaptations that were correlated with performance gains and health (Kyröläinen et al. 2004; Gillen and Gibala 2013), we hypothesized that this form of exercise may promote ME among older individuals.

Consequently, the aim of this study is to examine the effects of HIIT on ME values in sedentary young and older adults and to determine whether changes in fitness parameters such as maximal power are associated with ME changes. From a practical point of view, no study has determined and compared ME values by classifying the intensity of exercise among young and older participants. In fact, ME has been performed for the same workload. Therefore, this study evaluates ME using the ventilator thresholds as references to classify the intensity of incremental exercise.

## Materials and Methods

Seventeen healthy young adults [five males, 12 females, 26.2(2.4) years] and thirteen healthy older adults [five males, eight females, 54.5(2.3) years] were recruited from the Moncton campus of the University of Moncton. According to Canadian guidelines for body weight classification in adults, the younger participants were overweight ([BM = 82.3(3.8) kg, BMI = 28.6(1.1) kg/m^2^, FM = 27.1 (1.1) kg] and the older ones were obese [BM = 90.1(2.8) kg, BMI = 31.9 (1.1) kg/m^2^, FM = 33.8 (2.6) kg]. In order to better characterize our sample in this study, the age range was classified according to growth stages: young (18–40 years) and old adults (41–71 years).

Announcements were posted throughout the University campus and we invited students and staff who met the inclusion criteria to voluntarily participate in the project. The study protocol was approved by the University's Human Research Ethics Committee (UHRC), and all procedures were followed in accordance with the Helsinki Declaration of 1975, as revised in 2008. Informed consent was obtained from all subjects prior to inclusion in the study. The inclusion criteria for participation were as follows: participants had to be sedentary (<60 min.week^−1^ of structured exercise, as assessed by the International Physical Activity Questionnaire (Craig et al. 2003)), and none of them took part in any systematic exercise training at the time of study enrollment or during the 6 months that preceded the experiment; moreover, they had no history of orthopedic, neurological, cardiovascular or other chronic disease; no history of drug consumption before the study; and no history of smoking. During the first visit, before entering our protocol, each participant was thoroughly familiarized with the testing equipment and procedures for an hour. In this study, to reduce and control the potential effect of ovarian hormones on substrate metabolism in women, we controlled for the phase of the menstrual cycle (follicular phase) and the consumption of oral contraceptives. From an operational point of view, we managed that the women performed the incremental maximal test during the two visits at the same follicular phase period to avoid any hormonal interaction during the determination of energy expenditure and mechanical efficiency.

Next, the protocol began with two sessions of preliminary testing to determine baseline levels of key variables (anthropometric and fitness data, oxygen consumption, mechanical efficiency, energy consumption). The testing was conducted on two different days (Day one ‐ D1 and Day two ‐ D2) after an overnight fast, and took place in the morning of each day (~8 h 30). The two testing days were separated by a minimum of 48 h, and all subjects were asked to avoid physical activity for 48 h prior to each session. Throughout the study period the participants were asked not to consume alcohol and were instructed to continue their normal diet and maintain their typically sedentary behavior before intervention in order to not affect the variables measure. However, either physical activity or energy intake throughout the training period was not directly controlled during the study, then we cannot be sure if our instructions were fully respected. During the 6 weeks of intervention all participants completed all of the training sessions (thus adherence was 100%) and no other difficulties were encountered.

### Anthropometric measurements

Body mass, body fat percentage, fat‐free mass and fat mass were assessed using bioimpedancemeter (Bodystat1500, Isle of Man, British Isles). Height was determined to the nearest 0.5 cm with a measuring tape affixed to the wall. Body mass index (BMI) was calculated as the ratio of mass (kg) to height squared (m^2^).

### Physiological assessments

At baseline on D1 after a 12‐h overnight fast, each participant performed an incremental maximal test on a cycle ergometer (Monark ergomedic 839E electronic test cycle, Sweden) with continuous measurement of pulmonary gas exchange using a breath‐by‐breath automated metabolic system (Ergocard MEDI‐SOFT, Sorinnes, Belgium) to determine oxygen consumption (*V*O_2_max). Before beginning the test, participants remain seated for 5 min on the bicycle ergometer in the same position as that used for exercise. Resting oxygen consumption was based on mean oxygen consumption of the last 30 sec of minutes 3, 4, and 5. The test began at an initial power of 25 Watts and increases by 25 Watts every 5 min until exhaustion. During the test, participants were instructed to pedal at a rate of 50–70 revolutions per minute. The test was terminated when the participant requested to stop the exercise or could no longer maintain the required pedaling rate (revolutions per minute <40). A recovery phase of 5 min at 25 Watts followed the test (Jabbour et al. 2016).

In the present study, ventilatory thresholds were determined using established criteria as per (Wasserman et al. 1999) and used to classify the intensity of aerobic exercise. Briefly, VT1 corresponds to the break point in the plot of *V*CO_2_ as a function of *V*O_2_. At that point, VE/*V*O2 increases without an increase in VE/*V*CO2. VT2 was located between VT1 and *V*O_2_max when VE/*V*O_2_ begins to increase and VE/*V*O_2_ continues to increase. VT1 and VT2 were determined independently by three experienced investigators. To determine the *V*O_2_ and VE at VT1 and VT2, the average of the last 20 sec of each corresponding level was used.

Calibration was performed prior to each test using standard gases of known oxygen and carbon dioxide concentrations as well as a calibration syringe. The data were averaged over 30‐sec intervals, and both oxygen uptake and respiratory exchange ratios were measured. Maximal oxygen consumption, *V*O_2max_ (ml·kg^−1^·min^−1^), was achieved when a participant fulfilled at least three of the following criteria: a plateau in *V*O2 despite an increase in exercise intensity, a respiratory exchange ratio greater than 1.0, a maximal HR (continuously measured using a CASE 16 exercise testing system, Marquette, Wisconsin, USA) above 90% of the predicted maximal theoretical heart rate, HR (220 – age in years) or apparent exhaustion (Spiro 1977). All participants satisfied this requirement.

On D2, after 48 h rest, following 10 min of warm‐up, the selected participants performed a Force‐Velocity test on a cycle ergometer using a technique adapted from the study of (Vandewalle et al. 1988). This test consists of a succession of supramaximal bouts of approximately 6 sec, with flywheel resistance increasing by 1 kg after each episode until the subject is unable to perform the test. A period of passive recovery (5 min) was allowed between successive episodes. The peak velocity for each episode was recorded, and the power output was calculated by multiplying the load with the speed. The optimal load corresponded to the load at which maximal power (Pmax) was achieved. This load was then used for the HIIT protocol that followed. The Force‐Velocity test was also performed every 2 weeks to adjust the individual power level of supramaximal cycling exercise (SCE). The 2 days of testing took place before the HIIT (baseline testing), and again at the end of the training period (post intervention) following the same procedures.

### High‐intensity interval training

Once participants completed the preliminary testing, a total of 18 training sessions was prescribed (three sessions per week for 6 weeks) and performed in the laboratory room. The laboratory temperatures were kept stable (20 ± 2°C). The same training protocol has been previously developed and tested by our laboratory (Jabbour and Iancu 2015; Jabbour et al. 2015). Each of the prescribed sessions began with a 5 min warm‐up consisting of continuous cycling at moderate intensity corresponding to 40–50% of each participant's HRmax, and was followed by six repetitions of SCE intervals with 2 min of passive recovery between each repetition. Each SCE repetition lasted 6 sec, and participants were asked to pedal at maximal velocity against the resistance that was determined on D2. Heart rate values were monitored during all training sessions using a heart rate monitor (Kempele, Finland). The total duration of each session was approximately 16–18 min. During the training sessions, the velocity (in RPM) was recorded for each second of the entire round to ensure that participants pedaled at their maximal capacity. After concluding the 6 weeks of training, participants were asked to return for the final days of testing (post intervention), when the procedures of D1 and D2 were repeated and post‐training data were collected.

### Energy expenditure and mechanical efficiency determinations

*V*O_2_net was obtained by subtracting resting oxygen consumption from total oxygen consumption at each exercise stage. The net energy expenditure (EE) in Watts was calculated as follows (Garby and Astrup 1987). (4.94 · respiratory exchange ratio + 16.04) · (*V*O_2_net, in ml·min^−1^) · 60^−1^. Mechanical efficiency (ME) was also calculated in net terms as follows (Lafortuna et al. 2006): work produced in Watts • (E net, in Watts^−1^) · 100^−1^. EE and ME were obtained at intensities corresponding to VT1, VT2 and at *V*O_2max_. This method allowed us to compare these variables in terms of relative exercise intensity.

### Statistical analysis

Before analysis, all data were tested for normality (Kolmogorov–Smirnov test). For normally distributed data, inter‐ and intra‐group comparisons of the variables were computed by two‐way ANOVA (group *vs*. time) with repeated measurements to determine the main and interaction effects between groups over time. Pearson correlations were used to assess the association between changes in ME values and changes in fitness and anthropometry parameters. Multiple linear regression with an extended‐model approach was subsequently used to document the effects of the ME changes on selected variables. As a result, a series of multiple linear regression models were built for each anthropometric measurement and fitness level to determine the relationship between each variable and changes in ME. The analyses were performed using IBM SPSS Statistics 19 software (IBM SPSS Statistics for Windows, Version 24.0. Armonk, NY: IBM Corp.). A value of *P *<* *0.05 was considered statistically significant for all tests.

## Results

Subject characteristics are presented in Table 1. At baseline, body mass and BMI were significantly higher among older adults compared to young ones. Following 6 weeks of HIIT, body mass decreased only in the older group compared to baseline (Table 1) mainly due to a loss of FM. In addition, absolute maximal power output in Watts and relative maximal power in Watts per kilogram were significantly higher in both groups after HIIT compared to baseline (*P* < 0.01, respectively), and were higher in younger adults (620 W and 8.5 W.kg^−1^, *P* < 0.01, respectively) compared to older ones (600 W and 7 W.kg^−1^) (Table 1).

**Table 1:** Anthropometric and fitness data before and after HIIT

	Preintervention (baseline)		Postintervention		Group*Time effects	
	Young adults *(n = 17) W = 12, M = 5*	Older adults *(n = 13) W = 8, M = 5*	Young adults *(n = 17) W = 12, M = 5*	Older adults *(n = 13) W = 8, M = 5*	*F*	*P*
Age *(year)*	26.2 (2.4)	54.5 (2.3)^a^	–	–	9.8	<0.01
Height *(m)*	1.69 (1.1)	1.68 (1.3)	1.71 (1.1)	1.68 (1.3)	1.6	0.33
Body mass *(kg)*	82.3 (3.8)	90.1 (2.8)^a^	82.2 (1.1)	87.1 (3.3)^a^^,^ b	11.6	<0.01
BMI *(kg.m* ^*−2*^ *)*	28.6 (1.1)	31.9 (1.1)^a^	28.7 (1.4)	30.5 (1.5)^a^^,^ b	12.7	<0.01
FM *(kg)*	27.1 (1.1)	33.8 (2.6)	26.4 (1.7)	31.8 (2.9)^a^^,^ b	20.1	<0.01
FFM *(kg)*	56.1 (2.1)	56.2 (1.1)	57.1 (2.1)	56.1 (1.3)	0.4	0.63
Pmax *(W)*	520 (5)	540 (5)	620 (5)^b^	600 (6)^a^^,^ b	12.8	<0.01
Pmax *(W.kg* ^*−1*^ *)*	6.1 (0.3)	6.1 (0.5)	8.5 (1)^b^	7.1 (0.5)^a^^,^ b	12.8	<0.01
*V*O_2_max *(l.min* ^*−1*^ *)*	2.2 (1.1)	2.3 (0.5)	2.3 (0.5)	2.3 (0.5)	0.4	0.61
*V*O_2_max *(mL.min* ^*−1*^ *.kg* ^*−1*^ *)*	27.7 (3.6)	26.7 (2.1)	28.2 (3.5)	26.7 (3.5)	1.2	0.29

Values are mean ± SE (standard error). ***W,*** women; ***M,*** men; ***BM**,* Body Mass; ***BMI**:* Body Mass Index; ***FM***, Fat Mass; ***FFM**,* Fat Free Mass; ***VO***_***2max***_ ***,*** maximal oxygen consumption.

a Significant difference between groups (*P* < 0.01).

b Significant difference from baseline values (*P* < 0.01).

At rest and at VT2, oxygen consumption was significantly higher in the older compared to the younger group at baseline (Table 2). In contrast, maximal oxygen consumption did not differ statistically between groups. Following HIIT, *V*O_2_ did not differ between groups at rest and at VT1 and VT2 levels. No difference in respiratory exchange ratio was detected between the two groups at rest and at VT1 and VT2 levels at baseline and following HIIT training (Table 2).

**Table 2:** Mean values of oxygen consumption, mechanical efficiency, energy consumption, and respiratory exchange ratio during a graded exercise test before and after HIIT

	Preintervention (baseline)		Postintervention			
	Young adults *(n = 17) W = 12, M = 5*	Older adults *(n = 13) W = 8, M = 5*	Young adults *(n = 17) W = 12, M = 5*	Older adults *(n = 13) W = 8, M = 5*	Group*Time effects	
*Rest*						
*V*O_2_ *(ml.min* ^*−1*^ *)*	616 (2)	730 (5)^a^	580 (8)^b^	610 (6)^a^^,^ b	11.8	<0.01
RER	0.77 (0.01)	0.76 (0.02)	0.78 (0.02)	0.71 (0.01)	1.6	0.33
EE *(W)*	203 (13)	240 (25)^a^	190 (12)^b^	210 (17)^a^^,^ b	21.8	<0.01
Intensity corresponding to ventilator threshold 1 (VT1)						
HR *(beats.min* ^*−1*^ *)*	110 (4)	105 (3.5)	100 (3)	105 (3)	1.1	0.23
*V*O_2_ *(ml.min* ^*−1*^ *)*	1149 (63)	1168 (70)	1045 (63)^b^	1140 (60)^b^	21.2	<0.01
RER	0.84 (0.02)	0.85 (0.02)	0.81 (0.01)	0.81 (0.01)	1.6	0.33
EE *(W)*	183 (30)	170 (30)	169 (30)^b^	160 (10)^b^	11.8	<0.01
ME *(%)*	22.5 (2.1)	22.1 (0.8)	25.8 (2.1)^b^	26.1 (1.8)^b^	24.6	<0.01
Intensity corresponding to ventilator threshold 2 (VT2)						
HR *(beats.min* ^*−1*^ *)*	151 (4)	135 (6)	145 (4)	124 (5)	1.1	0.23
*V*O_2_ *(ml.min* ^*−1*^ *)*	1540 (91)	1717 (114)^a^	1634 (95)	1690 (136)	11.2	<0.01
RER	0.9 (0.01)	0.9 (0.02)	0.9 (0.01)	0.9 (0.02)	1.1	0.22
EE *(W)*	333 (30)	360 (23)^a^	320 (23)^b^	316 (28)^b^	21.5	<0.01
ME *(%)*	24.1 (1.1)	20.0 (1.3) a	25.9 (1.2)^b^	25.1 (1.3)^b^	22.6	<0.01
Workload at *V*O_2_max						
*V*O_2_ *(ml.min* ^*−1*^ *)*	2221 (485)	2338 (511)	2278 (580)	2298 (380)	3.1	0.63
RER	1.1 (0.1)	1.1 (0.1)	1.1 (0.1)	1.1 (0.01)	1.2	0.11
EE *(W)*	706 (45)	705 (50)	600 (45)^b^	610 (50)^b^	11.1	<0.01
ME *(%)*	25.5 (0.9)	23.2 (2.2)^a^	27.6 (0.8)^b^	27.1 (1)^b^	23.3	<0.01

Values are mean ± SE (standard error). ***HR***, heart rate; ***RER,*** respiratory exchange ratio; ***VO***_***2***_, oxygen consumption**;** ***EE*** **,** energy expenditure; ***ME,*** mechanical efficiency.

a Significant difference between groups (*P* < 0.01).

b Significant difference from baseline values (*P* < 0.01).

At baseline, EE, an indicator that takes oxygen consumption and respiratory exchange ratio into account, was statistically significantly higher in older adults at rest compared to younger ones (*P* < 0.01). Following HIIT, no differences remained between resting EE values. EE determined at VT1, VT2 and at *V*O_2max_ (Table 2) decreased significantly post‐training compared to baseline values in both groups (*P* < 0.01, respectively).

ME measured at VT1, VT2, and at *V*O_2max_ is reported in Table 2. Following HIIT, ME at VT1, VT2, and at *V*O_2max_ levels did not differ among groups and were significantly higher compared to values obtained at baseline (*P* < 0.01, respectively). These increases ranged from +2.7% and +4% at VT1, +2% and 5.1% at VT2 and +2.1% and 4.1% at 100% of *V*O_2max_ for younger and older groups, respectively. In this study, a positive and statistically significant relationship was found between resting EE and oxygen consumption and body mass values at baseline in both groups (*r* = 0.7, *r* = 0.78 and *r* = 0.69, *P* < 0.05, respectively). The multiple linear regression analysis demonstrated that the increases in maximal power output contributed significantly to increases in ME over 6 weeks at VT1, VT2 and at 100% of *V*O_2max_. This model accounted, respectively for 28, 38, and 42% of ME increases.

## Discussion

At baseline, the oxygen consumption and energy expenditure calculated at rest in both groups were significantly higher in the older compared to the younger group. This result is in contrast with previous findings showing that *V*O_2_ resting and energy expenditure are significantly lower in the elderly (65–89 years) compared to young subjects of both sexes (Kwan et al. 2004). For these authors, oxygen consumption declines with aging, lead to reductions in energy expenditure, seemingly not a consequence of the reduced fat‐free mass. In addition, (Visser et al. 1995), and (Hunter et al. 2001) suggested that aging had an effect on energy metabolism (ex. reduced resting metabolic rate). However, it is important to mention that several factors were not considered in these works, such as the potential impact of comorbid conditions and physical activity levels known to affect energy expenditure and oxygen consumption among individuals. As for this study and according to Canadian guidelines for body weight classification in adults, the younger participants were overweight and the older ones were obese. As previously reported, greater energy expenditure at rest is a consequence of higher fat‐free mass and muscle mass (Muller et al. 2002), increased work breathing (Pelosi et al. 1996), and altered substrate utilization (Sun et al. 2004). In this study, fat‐free mass and respiratory gas exchange, which reflect substrate utilization, did not differ between groups at rest. However, the results showed a positive relationship between body weight and both oxygen consumption and energy expenditure at rest in both groups. These findings indicate that body weight seems to be of major importance in explaining higher energy expenditure in the older group. Following 6 weeks of HIIT, resting energy expenditure decreased significantly in the older group compared to baseline. According to this study's results, this decrease was concomitant with a decrease in resting oxygen consumption and was significantly associated with a decrease in body weight in older adults.

At VT1, EE and ME did not differ between younger and older subjects. These results differ from several works (Pearce et al. 1983; Ortega and Farley 2007) that have reported lower ME at all work stages. Before training, at high‐intensity levels (e.g., VT2 and VO_2_max), ME was significantly higher in the young compared to the older. At VT2 level, the lower ME values observed for older adults may be related to energy expenditure increases given that ME depend on the amount of energy consumption for a given work performed (Weinstein et al. 2004). However, at *V*O2max level, the differences between the two groups may be attributed to the lower muscle efficacy to perform work given that at this intensity the energy expenditure values were similar between the two groups. This result is in accordance with previous data (Pearce et al. 1983; Ortega and Farley 2007). However, ME calculated during bicycle ergometer tests reported contradictory results. While Babcock et al. (1992) showed that elderly adults have similar exercise efficiency than younger ones, others have reported greater (Venturelli et al. 2013) or lower (Bell and Ferguson 2009) ME among older populations. Actually, many issues might limit generalization of results among older populations such as the use of a non‐steady‐state “ramp” protocol (Venturelli et al. 2013), a failure of considering body weight statuses (Ortega 2013; Venturelli et al. 2013) and mobility impairments due to a combination of aging and specific disease conditions (Hoffman et al. 1996). Additionally, from a practical standpoint, no study has determined and compared ME values by classifying the intensity of exercise among participants. In fact, ME has been performed for the same workload; therefore, it is unwarranted that said workload corresponds exactly in terms of relative work among participants. To avoid this incertitude, we have used the ventilator thresholds as references to classify the intensity of incremental exercise. According to this study's results, VT1 corresponds to ~ 45% of *V*O_2_max and VT2 corresponds to ~60% of *V*O_2_max. Therefore, it seems difficult to establish a general statement as for the relationship between ME and age base.

Following 6 weeks of HIIT, maximal oxygen uptake did not change in either group of subjects. Maximal power output did increase in both groups (+100 W for young and 60 W for older individuals) despite no concomitant change in fat‐free mass. For ME, the values determined at VT1, VT2 and at 100% of *V*O_2_max increased significantly in both groups, compared to baseline.

Interestingly, the ME observed in this study in the older group, which reflects the muscle efficacy to perform work, increased approximately two times more than in the young group. To the best of our knowledge, no data has been reported regarding ME values in response to interventions in older populations. According to our previous data, improvements in ME observed in response to HIIT could be attributed to improvements in the metabolic milieu (HOMA‐IR), without excluding increases in muscle performance (Jabbour and Iancu 2015). In fact, alterations in metabolic milieu may impair substrate use (e.g., carbohydrate) and muscle performance; therefore, HIIT may improve these parameters (Jensen et al. 1987; Dawson et al. 1998). In the current study, our results indicate that ME increases observed in both groups correlated positively with maximal power output increases. The latter was identified as significant predictors of ME increases over 6 weeks at VT1, VT2 and at 100% of *V*O_2_max, accounting respectively for 28, 38, and 42%, regardless of the relationship. Therefore, without excluding increases in muscle performance (e.g., muscle power), further studies are needed to establish the relationship between metabolic milieu and ME improvements among older individuals.

## Conclusion

To the best of our knowledge, this study is the first to examine ME changes in response to 6 weeks of HIIT in older and younger adults. Our analyses reveal that at baseline, the ME values calculated at lower intensities (VT1) did not differ among groups; however, at higher intensities (VT2 and at *V*O_2max_), ME values were significantly lower in older than in younger adults. These lower ME values were concomitant to increases in energy expenditure observed in older adults. However, there was a significant increase in ME levels in response to HIIT, in both groups, changes associated with improved energy expenditure, and increases in power output. Our results suggest that HIIT can be recommended as strategy aimed at improving muscle efficiency in older adults.

## Conflict of Interest

The authors declared no conflict of interests regarding the publication of this manuscript.
